# Noise-induced speed up in repetitively firing neurons occurs far from spike threshold

**DOI:** 10.1186/1471-2202-15-S1-P11

**Published:** 2014-07-21

**Authors:** Todd W Troyer, David Barraza, Michael A Farries, Charles J Wilson

**Affiliations:** 1 Biology Department, University of Texas, San Antonio, TX 78249, USA; 2 Dept. of Psychology, University of Michigan, Ann Arbor, MI, 48109, USA

## 

Injecting white noise currents has been shown to increase mean firing rate in several types of neuron. This speed up is generally attributed to the fact that depolarizing currents on the tails of the noise distribution push cells past spike threshold sooner than they would have otherwise. As part of a larger effort to understand the dynamic properties of rat subthalamic (STN) neurons, we have performed perforated patch recordings of these tonically active neurons in brain slices, exposing them to contiguous sequences of brief current pulses drawn from a Gaussian distribution. Pulsed noise disrupted firing pattern with no significant change in rate over a wide range of noise amplitudes, so long as the pulse durations were brief (<2 ms). With longer pulse durations, neurons increased their firing rate with noise, with greater increases for pulses of larger magnitude and longer duration. However, individual neurons displayed varying degrees of noise-induced speed up.

We used phase resetting methods to understand the noise-induced changes in the mean and variance of the recorded interspike interval distributions. For each cell, a phase response curves (PRC) was derived by using linear interpolation to estimate the phase of the neuron between spikes, followed by a linear regression to estimate the sensitivity of the cell to input perturbations at each phase. We then compared Monte Carlo simulations of a one-dimensional phase model to predict the response patterns of individual neurons. Surprisingly, simulation results produced a good match to experimental mean and variances even at noise levels leading to autocorrelation histograms with no visible periodicity (CVs ~ 0.4).

We then investigated simulations results in detail to understand the dynamic mechanism that underlies the noise-induced speed up in these neurons. Plots of the mean phase as a function of time revealed that the noise-induced phase advance occurred early in an interspike interval, well before trajectories approach the time of the next spike. This phase advance can be understood as ‘noise-induced drift’ in the Stratanovich interpretation of stochastic differential equations, with possible supralinear contributions for pulses of finite duration. Current research uses artificially constructed PRCs to examine how the increasing spread of the phase distribution during each intervals leads to an asymmetric sampling of the noise-induced drift, preventing the early noise-induced speed up from being canceled by an expected noise-induced slowing as the PRC approaches zero near the time of the next spike.

